# Forty-five years of schizophrenia trials in Italy: a survey

**DOI:** 10.1186/1745-6215-13-35

**Published:** 2012-04-12

**Authors:** Marianna Purgato, Clive Adams, Corrado Barbui

**Affiliations:** 1 Department of Public Health and Community Medicine, Section of Psychiatry, University of Verona, Verona, Italy; 2 Division of Psychiatry, University of Nottingham, Nottingham, UK; 3 Department of Public Health and Community Medicine, Section of Psychiatry, University of Verona, Piazzale L.A. Scuro, 10-37134 Verona, Italy

**Keywords:** schizophrenia, psychopharmacology, quality, randomized controlled trials, Italy

## Abstract

**Background:**

Well-designed and properly executed randomized controlled trials (RCTs) provide the best evidence on the efficacy of healthcare interventions. Mental health has a strong tradition of using trial to evaluate treatments, but the translation of research to clinical practice is not always easy. Even well-conducted trials do not necessarily address the needs of every day care and trials can reflect local needs and the specific culture in which they are undertaken. Generalizing results to other contexts can become problematic but these trials may, nevertheless, be very helpful within their own context. Moreover, pathways for drug approval can be different depending on local regulatory agencies. Local trials are helpful for decision-making in the region from which they come, but should not be viewed in isolation. National quantity and quality of trials may vary across nations.

The aim of this study is to quantify trialing activity in Italy from 1948 until 2009 and to describe characteristics of these trials. In addition, we evaluated change over time in three keys aspects: sample size, follow-up duration, and number of outcomes.

**Methods:**

We used the Cochrane Schizophrenia Group's register that contains 16,000 citations to 13,000 studies relating only to people with schizophrenia or schizophrenia-like illness. Randomized controlled trials and controlled clinical trials undertaken in Italy and involving pharmacological interventions were included.

**Results:**

The original search identified 155 records of potentially eligible studies, 74 of which were excluded because do not meet inclusion criteria. A total of 81 studies were included in the analysis. The majority of trials were conducted in north Italy, and published in international journals between 1981 and 1995. The majority of studies (52 out of 81) used standardized diagnostic criteria for schizophrenia disorder. They were defined as randomized and used blind methods to administer treatment. However, most failed to report detail regarding methodological procedures and it is difficult to ascertain which studies are associated with a low risk of bias.

**Conclusions:**

Trials should be designed to address the needs of everyday care with the aim of following large samples of typical patients in the long term. The Italian tradition in the area of trialing treatments for people with schizophrenia is not as strong as in many other similar countries and Italy should be producing more, better, independent, and clinically relevant trials.

## Background

Good mental health research is critical to guide healthcare professionals to make informed decisions about the effects of most interventions. Early examples do exist of attempts at fair evaluation of healthcare, including several from Italy [1,2]. However, in 1948 the UK MRC Streptomycin randomized trial was published and remains a landmark of modern healthcare evaluation [3]. After that many specialties began to adopt randomization for the evaluation of treatments.

Mental health has a strong tradition of using trials [4], but translation of research to clinical practice is not always easy. For patients, carers, and policymakers, local data are important. Even well-conducted trials, if undertaken in a very dissimilar care-culture may be difficult to apply. Local trials are important and informative whether they concur - or contradict - other similar studies from afar. The totality of evidence must be considered but the local perspective not ignored. Not to have local studies leaves all interested in the effects of care vulnerable to importing data of limited applicability. Some nations, however, have produced few trials. For schizophrenia trials, measures of national wealth and not public health burden loosely predict the research activity of a country [5]. Italy, however, is the 10th most wealthy nation in terms of GDP http://www.nationmaster.com/graph/eco_gdp-economy-gdp.

The quality of mental health research, and trials in particular, may also vary across nations [4]. For example, the volume and quality of trial research from China has been considered in many surveys and quality remains a major concern [6-10]. Elsewhere it has been shown that pioneering mental health trials from low and middle income countries are of as mixed quality as their more accessible counterparts from richer nations but cannot be identified in commonly used bibliographic databases [11]. Nearer home, in Europe, Romania's mental health research has been the focus of recent investigation and the increasing dominance of pharmaceutical industry noted [12].

The Cochrane Schizophrenia Group produces and maintains a register of all studies http://szg.cochrane.org/cochrane-schizophrenia-group-specialised-register. This involves regular and systematic searching of 71 databases. The studies identified in this way are reliably indexed by country. The aim of this study is to use part of this dataset to quantify trialing activity in one area of mental healthcare over time in Italy and describe characteristics of these trials. Specifically, we evaluated content and risk of bias of Italian trials relevant to people with schizophrenia from 1948 until 2009 for three key methodological aspects: sample size, duration, and number of outcomes.

## Methods

### Source

We searched the Cochrane Schizophrenia Group Register without time limitation. The search was made in May 2011 and the last version of register was updated in April 2011. The register includes all published and unpublished references to randomized, quasi-randomized and controlled clinical trials without language restrictions. The register is maintained on Meerkat 1.6. The Cochrane Schizophrenia Group's register contains 16,000 citations to 13,000 studies relating only to people with schizophrenia or schizophrenia-like illness http://szg.cochrane.org/cochrane-schizophrenia-group-specialised-register. These studies are reliably indexed regarding the country of origin, the interventions under study, and the number of participants.

### Types of studies

We included all randomized controlled trials and controlled clinical trials undertaken in Italy. Studies were included if any pharmacological treatment was compared with other active pharmacological treatments or placebo. Only studies that enrolled patients in Italy were considered. Multicenter studies were included if all centers enrolling patients were located in Italy.

### Selection of trials and data extraction

From the Cochrane Schizophrenia Group Register we extracted all records corresponding to studies carried out in Italy. We examined all titles and abstracts, and obtained full texts if the word 'random' or 'randomized' or 'control' or 'controlled' was present in the title and/or abstract. MP read the full texts, determined whether they met inclusion criteria, and extracted the data. Data were extracted using an electronic spreadsheet. Considerable care was taken to exclude duplicate publications. In order to ensure consistency, CB and CEA carried out a reliability check on all data extracted by comparing the data abstracted in the electronic spreadsheet with the paper version of each study. In case of disagreement between reviewers this was resolved by discussion.

We considered the following variables: year of publication, geographic area (north, center, south of Italy), language of publication (English, Italian), pharmacological treatment (Antipsychotics according to the WHO Anatomical Therapeutic Chemical [ATC] classification system, other pharmacological treatments), sample size, weeks of follow-up, diagnostic criteria (standardized criteria such as DSM or ICD, implicit criteria such as clinical judgment, or unclear criteria), description of random allocation and blinding (using the Cochrane Collaboration 'Risk of BIAS' tool criteria), number of outcome measures (number of different psychopathological dimensions analyzed, as indexed in the specialized register), and use of the CONSORT flow-diagram.

### Data presentation

We calculated simple percentages (%) with 95% confidence intervals (CI). To ascertain whether sample size, length of follow-up, and number of efficacy measures have increased in the last 45 years, we used a box plot diagram and a non-parametric test for trend (extension of the Wilcoxon rank-sum test). Spearman's rank correlation coefficients for all pairs of variables were additionally calculated using data in continuous format. STATA 11 was used to carry out the statistical analysis.

## Results

### General characteristics

**Figure 1:**
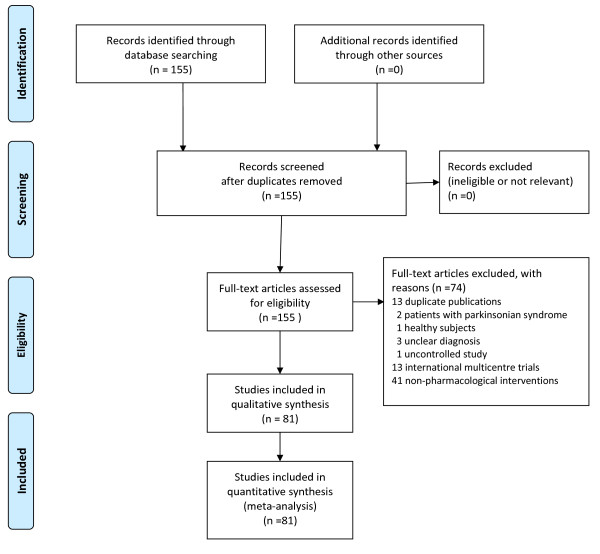
**PRISMA flow diagram**.

The original search identified 155 records of potentially eligible studies, 74 of which did not meet inclusion criteria (Figure 1). For the remaining 81 records full text was retrieved and data extracted. The characteristics of included studies are presented in Table 1.

**Table 1:** Characteristics of schizophrenia drug trials published in Italy from 1948 to 2009 (*n *= 81 studies)

	(*n*)	% (95% CI)
*Area of Italy*		
North	42	51.8 (40.4-63.0)
Center	19	23.4 (14.7-34.1)
South	20	24.6 (15.7-35.5)
Year of publication*		
1948-1980	20	25.3 (16.2-36.3)
1981-1995	36	45.5 (34.3-57.1)
1996-2009	23	29.1 (19.4-40.4)
*Language of publication*		
English	58	71.6 (60.4-81.0)
Italian	23	28.4 (18.9-39.5)
*Drug treatment*		
Antipsychotics	64	79.0 (68.5-87.2)
Other drugs	17	20.1 (12.7-31.4)
*Diagnostic criteria*		
Unclear	3	3.7 (0.77-10.4)
Standardized criteria	52	64.2 (52.7-74.5)
Implicit criteria	26	32.1 (22.1-43.3)

*Two unpublished studies not included

*CI *confidence interval.

Most studies (52% CI 40-63) were conducted in north Italy, the majority were published between 1981 and 1995 (46% CI 34-57), and 72% (CI 60-81) were in English. The majority of studies evaluated antipsychotic drugs (79%, CI 69-87), while a minority assessed the beneficial effect of other drug treatments. Most also used standardized diagnostic criteria for defining schizophrenia (64% CI 53-75), whereas 32% (CI 22-43) used implicit criteria and 4% (CI 1-10) do not specify any diagnostic criteria.

### Methodological characteristics

**Figure 2:**
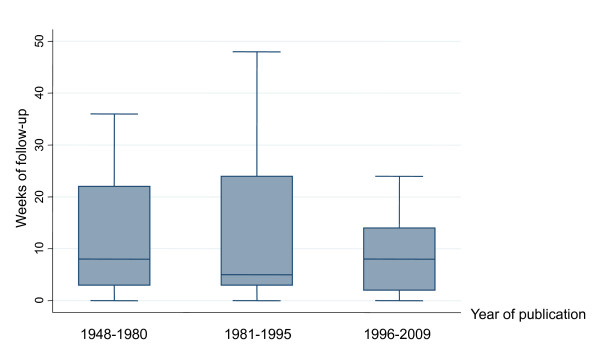
**Length of Italian schizophrenia drug Trials published between 1984 and 2009 (*n *= 81)**. The horizontal line represents the median, the box extends to cover the interquartile range and the vertical line extends to the extremes.

Most trials were of short duration, with only 20 (24.6% CI 15.7-35.5) being of medium or long-term follow-up (13 weeks or more) (Table 2). In 10 cases the length of follow-up was unclear. Duration does not increase over time (z for trend = -0.41, *P *= 0.685) (Figure 2). Spearman's rank correlation coefficient confirmed no association between year and length of follow-up (rho = -0.030; *P *= 0.814)

**Table 2:** Main methodological characteristics of schizophrenia drug trials published in Italy from 1948 to 2009 (*n *= 81 studies)

	*n*	% (95% CI)
*Sample size*		
Min-20	22	27.1 (17.8-38.1)
21-40	32	39.5 (28.8-50.9)
40-max	27	33.3 (23.2-44.6)
*Length of follow-up (weeks)*		
Unclear	10	12.3 (6.08-21.5)
2-4	30	24.6 (15.7-35.5)
5-12	21	25.9 (16.8-36.8)
13+	20	24.6 (15.7-35.5)
*Randomization*		
Randomized, no details	51	62.9 (51.5-73.4)
Randomized with details	7	8.6 (3.54-16.9)
Unclear	23	28.4 (18.9-39.5)
*Blinding*		
Single blind	6	7.4 (2.76-15.4)
Double blind	47	58.0 (46.5-68.9)
Unclear	28	34.5 (24.3-45.9)
*Outcome measures (n)*		
1-5	33	40.7 (29.9-52.2)
6-15	35	43.2 (32.2-54.6)
16-60	13	16.0 (8.83-25.8)
*Patient flow diagram*		
Yes	2	2.4 (0.30-8.63)
No	79	97.6 (91.3-99.6)

*CI *confidence interval

**Figure 3:**
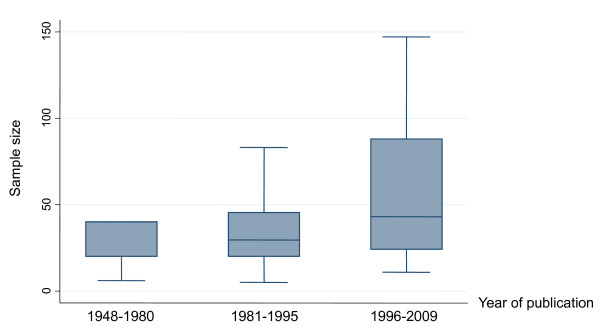
**Number of the patients in Italian schizophrenia drug trials published between 1984 and 2009 (*n *= 81)**. The horizontal line represents the median, the box extends to cover the interquartile range and the vertical line extends to the extremes.

Two-thirds of trials had less than 40 participants (67% CI 55-77); sample size only minimally increased over time (Figure 3), as shown by Spearman's rank correlation coefficient (rho = 0.259; *P *= 0.020) but not by test for trend (z for trend = 0.48, *P *= 0.628). While the use of standardized diagnostic criteria progressively increased, accounting for more that 70% of trials published after 1995 (17/23), the use of implicit criteria dropped, accounting for less than 20% of trials published after 1995 (4/23).

Most trials were described as 'randomized' even though only seven (9% CI 4-17) provided details about the methods of allocation (Table 2). In 23 studies (28% CI 19-40) the allocation procedure was unclear. Single blind was adopted in six studies (7% CI 3-15), double blind was adopted in 47 studies (58% CI 47-69). For the remaining 28 studies (35% CI 24-46) it was unclear if blinding was used or not.

**Figure 4:**
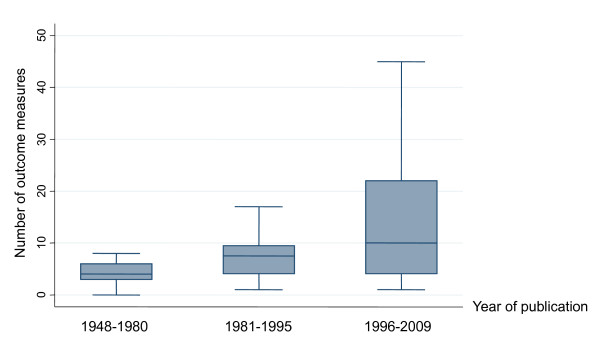
**Number of outcome measures employed in Italian schizophrenia drug trials published between 1984 and 2009 (*n *= 81)**. The horizontal line represents the median, the box extends to cover the interquartile range and the vertical line extends to the extremes.

The number of outcomes measures ranged from 1 to 5 in 33 studies (41%, CI 30-52), from 6 to 15 in 35 trials (43%, CI 32-55) and from 16 to 60 in 13 studies (16% CI 9-26). Numbers of outcomes measures increases over time (z for trend = 3.32, *P *= 0.001) (Figure 4). Spearman's rank correlation coefficient confirmed the association between year and number of outcomes (rho = 0.436; *P *< 0.001).

The CONSORT statement was first published in 1996 [13]. Two of the 23 studies since that time reported a patient flow diagram (8% CI 1-28).

## Discussion

To our knowledge, this is the first survey investigating content and volume of trial activity in Italy. Although the majority of Italian schizophrenia trials were published in international journals, most failed to report basic methodological details such as, for example, information about the methods of random allocation, and its concealment from the study investigators, or how blinding was preserved. It is therefore difficult to ascertain which studies are associated with a low risk of bias. This compelling issue similarly applies to trial activity conducted in other countries. China, for example, a country which has progressively increased its production of randomized studies in the last 10 years, is still under scrutiny for some aspects of trial quality, including random allocation, blinding, and dropout reporting. This seems quite relevant in different areas of medicine [14], as well as in mental health [15]. Poor reporting in mental health is common, involves both high and low income countries [4], and leads to risk in interpreting results [16]. In most cases it is not possible to go beyond identifying studies that seem to be randomized trials. This further reinforces the need to develop tools to better describe and appraise the adequacy of the randomization process in a culturally sensitive manner. Clearly, random allocation is a scientific process and it should not differ across cultures, but its reporting should take into consideration the different meaning that the world random might have in different cultures.

The Consolidated Standard of Reporting Trial (CONSORT) statement [13,17] facilitates complete and transparent reporting, aids critical appraisal and interpretation of results. This simple checklist for reporting has been widely adopted [18]. Good trial reporting is important not only for guiding clinicians towards correct decision-making, but also for regulatory agencies working on drug approval. Certainly, clinicians and regulatory authorities should require higher standards from clinical trial reports [19]. Researchers and editors no longer have an excuse for bad reporting.

In Italy, in this subspecialty, while sample size increased only minimally over time, patient selection criteria and outcome assessments have become much more sophisticated, as suggested by the increase in use of standardized diagnostic criteria and by the steadily increase in the number of outcome measures. Although this trend may have increased the internal validity of findings, it has nevertheless allowed study of only highly refined groups of people with schizophrenia. This increasing drift from real world practice makes it difficult to apply trial results to typical patients [20,21]. Similarly, we observed that the number of outcome measures has increased during the last 45 years. This confusion of measuring suggests, at the very least, a lack of consensus on what is important. Another example, if one was needed, supporting the need for a set of core outcome measures in this area [22]. Also increasing numbers of measures inevitably enhanced the probability of detecting chance significant differences. Rarely had the trials addressed this issue in the statistical analysis or in the interpretation of findings.

Our survey has some limitations. First, we did not include international multicenter studies where Italy was one site, possibly losing studies with good sample sizes and potentially better reporting. Second, as our focus was drug trials only, we collected no data on content and volume of trial activity in other fields of schizophrenia treatment, including psychological treatments, psychosocial interventions, and organizational approaches. This limits the generalizability of our findings, as we do not know if the current standard of drug trials can be considered representative of the whole spectrum of trial activity. Finally, we focused our survey on some indicators only, while it would have been of interest to describe trial activity with respect to other aspects, such as for example setting, intention to treat versus per protocol analysis, dropout reporting, economic support, ethics committee approval, and consent 'rituals'.

## Conclusions

The Italian tradition in the area of evaluating treatments for people with schizophrenia is not as strong as in other similar countries [5]. Perhaps Italy has relied too much on studies undertaken outside its borders. There is an opportunity to produce more, better, independent, and nationally and internationally clinically relevant trials.

## Competing interests

The authors declare that they have no competing interests that materially affect the contents of this work.

## Authors' contributions

MP, CEA, and CB designed the study. MP extracted data. MP, CB, and CEA analyzed and interpreted data. MP, CB, and CEA drafted the first manuscript. CB and CEA commented and refined the manuscript in preparation for submission. All authors approved the final version to be published.

## Funding

None of the authors received financial support for this work.
